# Associations Between Leisure‐Time Physical Activity and Metabolomics‐Based Markers of Biological Aging in Late Midlife: Short‐Term and Long‐Term Follow‐Up

**DOI:** 10.1111/acel.70033

**Published:** 2025-03-10

**Authors:** Katri Ruutu, Niko S. Wasenius, Kothandaraman Narasimhan, Tuija M. Mikkola, Merja K. Laine, Johan G. Eriksson

**Affiliations:** ^1^ Folkhälsan Research Center Helsinki Finland; ^2^ Faculty of Medicine University of Helsinki Helsinki Finland; ^3^ Department of General Practice and Primary Health Care University of Helsinki and Helsinki University Hospital Helsinki Finland; ^4^ Institute for Human Development and Potential Agency for Science, Technology and Research Singapore City Singapore; ^5^ Population Health Unit Finnish Institute for Health and Welfare Helsinki Finland; ^6^ Clinicum, Faculty of Medicine University of Helsinki Helsinki Finland; ^7^ Yong Loo Lin School of Medicine National University of Singapore Singapore City Singapore

**Keywords:** accelerometer‐based physical activity, biological aging, leisure‐time physical activity, metabolic aging, metabolomics

## Abstract

Physical activity (PA) may delay the onset of age‐related diseases by decelerating biological aging. We investigated the association between leisure‐time physical activity (LTPA) and metabolomics‐based aging markers (MetaboAge and MetaboHealth) in late midlife and during 16 years of follow‐up. At the 16‐year follow‐up, we also investigated the association between device‐based PA and MetaboAge and MetaboHealth. We included 1816 individuals (mean age 61.6 years) from the Helsinki Birth Cohort Study at baseline and followed them up for 5 (*n* = 982) and 16 years (*n* = 744), respectively. LTPA was assessed via questionnaire at baseline and 16 years later and device‐based PA with ActiGraph accelerometer at the 16‐year follow‐up. Fasting blood samples were applied to calculate MetaboAge acceleration (ΔmetaboAge) and MetaboHealth at baseline and at both follow‐ups. Covariate‐adjusted multiple regression analyses and linear mixed models were applied to study the associations. A higher volume of LTPA at baseline was associated with a lower MetaboHealth score at the 5‐year follow‐up (*p* < 0.0001 for time × LTPA interaction). No associations were detected at the 16‐year follow‐up. An increase in LTPA over 16 years was associated with a decrease in MetaboHealth score (*p* < 0.001) and a decrease in LTPA with an increase in MetaboHealth score. Higher device‐based PA was associated with a lower MetaboHealth score, but not with ΔmetaboAge. In conclusion, higher LTPA in late midlife and device‐based PA in old age were associated with improved MetaboHealth. Increasing LTPA with age may protect against MetaboHealth‐based aging. The results support the importance of PA for biological aging in later life.

## Graphical abstract

PA may delay the onset of age‐related diseases by decelerating biological aging. We found that higher self‐reported amounts of PA in late midlife and device‐based PA in old age were associated with improved MetaboHealth and that reaching twice the amount of the current PA recommendations in late midlife may lead to decelerated biological aging. In addition, increasing LTPA from late midlife to old age may protect against metabolomics‐based biological aging.

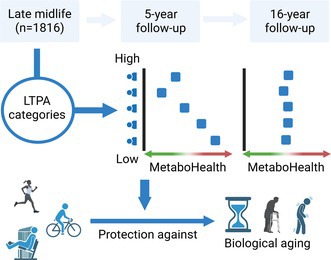

## 1 Introduction

The global population is aging rapidly, challenging not only the well‐being of individuals but also the social, economic, and demographic backbone of societies (World Health Organization 2015). Aging increases the risk for diseases, disability, and adverse clinical health outcomes (Kennedy et al. 2014). The challenges set by aging have sparked the search for novel biomarkers of aging using omics‐based data (Moqri et al. 2023; Rutledge et al. 2022).

Biomarkers of aging aim to quantify the level of age‐related biological changes expressed as a number, biological age, or as age acceleration describing the difference between chronological and biological age (Moqri et al. 2023). Dysregulation of metabolism has been recognized as one of the hallmarks of aging, also referred to as metabolic aging (López‐Otín et al. 2023) justifying the use of metabolomics‐based aging biomarkers in aging research. Aging biomarkers built using other types of omics data (epigenomic, transcriptomic, proteomic) have also been extensively used as measures of biological age, but they for the most part capture different aspects and hallmarks of aging (Rutledge et al. 2022). For example, epigenetic aging biomarkers that are based on DNA methylation values at specific CpG sites have only weak correlations with metabolomics‐based aging biomarkers, correlations ranging from −0.22 to 0.21 for MetaboAge and from 0 to 0.32 for MetaboHealth (Kuiper et al. 2023). The advantage of metabolomics‐based aging biomarkers compared to other omics‐based biomarkers lies in the fact that the metabolome carries more systemic information from multiple tissues across the body than, e.g., the methylome or transcriptome (Buergel et al. 2022). In addition, metabolomics‐based biomarkers are trained on large sample sizes (Rutledge et al. 2022), and in recent years, nuclear magnetic resonance (NMR)‐based metabolomics have matured and are now available at lower cost (Wishart et al. 2022). Recent advances in multi‐omics aging biomarkers show promise in capturing biological aging better than single omics‐based aging biomarkers. However, multi‐omics biomarkers are more complex and expensive and require more expertise, which limits their clinical feasibility (Mohr et al. 2024).

Recently, an NMR platform was used to develop metabolomics‐based biomarkers of aging (Soininen et al. 2015; Würtz et al. 2017) that are trained for chronological age (MetaboAge; van den Akker et al. 2020) and mortality (MetaboHealth; Deelen et al. 2019). These metabolomics‐based markers of biological age have been successfully applied to predict risk for age‐related diseases (e.g., cardiovascular diseases), comorbidities, and mortality (Deelen et al. 2019; Kuiper et al. 2023; van den Akker et al. 2020). Hazard ratio (HR) per unit increase for the prediction of mortality risk has been shown to be 1.25 (1.14–1.37) for MetaboAge (per year increase; van den Akker et al. 2020) and 2.73 (2.60–2.86) for MetaboHealth (per unit increase; Deelen et al. 2019). In other often employed omics‐based aging markers, the corresponding HRs per unit increase for the prediction of mortality risk have been, e.g., 1.10 (1.09–1.12) for GrimAge (per year increase; Lu et al. 2019), and 1.03 (1.02–1.04) for the proteomic biomarkers signature of age (per year increase; Tanaka et al. 2020). In a recent review of blood‐based composite biomarker validation studies, MetaboHealth stood out from other biomarkers of aging as an excellent potential candidate for the prediction of future mortality risk and for use in future clinical studies (Moqri et al. 2024). However, the interpretation of HRs between different omics‐based biomarkers should be done with caution, e.g., due to their different units of measure and population characteristics.

Promotion of physical activity is a well‐established strategy to tackle age‐related diseases and comorbidities (Kyu et al. 2016). A greater amount of physical activity has also been associated with lower mortality rates (Ekelund et al. 2019). Studies concerning physical activity and metabolomics‐based biomarkers of aging are scarce and partly contradictory. These studies have relied on self‐reported physical activity and have mainly been cross‐sectional, finding no association between physical activity and MetaboAge (Jansen et al. 2021), MetaboHealth (Smit et al. 2024), and a combined urine and serum metabolomics‐based age (Robinson et al. 2020). Some studies have found a significant inverse association between physical activity and a urine metabolomics‐based biological age score (Hertel et al. 2016) and MetaboHealth (Kuiper et al. 2024) in a cross‐sectional setting. These inconsistent findings could be attributed to differences in biosamples, methodologies, or the methods used to calculate metabolomics‐based biological age scores or differences in the study populations, such as age. Only one study has investigated the association between physical activity and metabolomics‐based biological aging in a longitudinal setting. In a study conducted by Kuiper et al. (2024) adherence to and starting to adhere to physical activity guidelines, but not the volume of physical activity, was inversely related to MetaboHealth among 55‐year‐old adults over a 10‐year follow‐up. Thus, we are currently lacking information on whether the associations reach over a 10‐year period, whether they hold among people approaching old age, and whether the present evidence is supported by data from device‐based physical activity.

Therefore, we used prospective data from the Helsinki Birth Cohort Study (HBCS) with a short‐term (5 years) and a long‐term (16 years) follow‐up and aimed to investigate the associations of the self‐reported leisure‐time physical activity (LTPA) with metabolomics‐based aging biomarkers (MetaboAge and MetaboHealth) in late midlife and their 5‐year and 16‐year changes. In addition, we investigated whether the change in LTPA over the long‐term follow‐up is associated with the change in MetaboAge and MetaboHealth over the years. We also performed supplementary cross‐sectional analyses between device‐based physical activity data and MetaboAge and MetaboHealth when participants were on average 75 years old.

## 2 Materials and Methods

### 2.1 Study Design and Participants

This prospective cohort study applied data from the HBCS between the years 2001 and 2018. The original HBCS includes a total of 13,345 individuals who were born between 1934 and 1944 at the Helsinki University Central Hospital or the Helsinki City Maternity Hospital and who visited child welfare clinics at that time in the city. Individuals who were born at Helsinki University Hospital and were still alive in 1971 when a unique personal identification number was given to the residents of Finland were identified from the original cohort, resulting in 8760 individuals. From that population, 2902 individuals who were still alive and living in Finland were randomly selected using random‐number tables and invited to the first clinical examination in 2001–2004. Of those 2902 invited individuals, 2003 individuals participated in the first clinical examination. The second clinical examination took place in 2007–2008, and it included individuals who were still alive and did not have diabetes at the first clinical examination. Altogether, 1083 individuals participated in the second clinical examination. The criteria for identifying individuals for the third clinical examination in 2017–2018 were still being alive and living within 100 km of the study clinic in Helsinki. In total, 1174 individuals were invited, and in the end, 815 individuals participated in the third clinical follow‐up visit. Longitudinal data until the follow‐up in 2017–2018 from the same cohort regarding frailty and the prevalence of healthy aging have previously been published (Haapanen et al. 2023; Mikkola et al. 2023), this study expanding on the role of physical activity from late midlife to old age in the metabolomics‐based biological aging.

**FIGURE 1:**
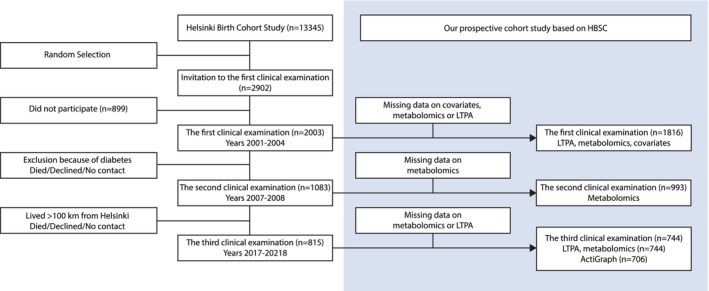
Study design.

In this study (Figure 1), we excluded participants who had missing information on covariates at the first clinical examination or on LTPA either at the first or the third clinical examination. At each clinical examination, we also excluded participants who had missing data on metabolomics. The final sample consisted of 1816 individuals at the first clinical examination, 993 individuals at the second clinical examination, and 744 individuals at the third clinical examination. Of the sample at the third clinical examination, device‐based physical activity data were available for 706 individuals.

The study was approved by the Ethics Committee of the Hospital District of Helsinki and Uusimaa and the Ethics Committee of the National Public Health Institute, Helsinki. Each participant signed the written informed consent before any study procedures were performed.

### 2.2 Leisure‐Time Physical Activity

We assessed LTPA twice, at baseline during the first clinical examination in 2001–2004 and in the clinical examination in 2017–2018. We assessed LTPA with a validated Kuopio Ischemic Heart Disease (KIHD) 12‐month recall questionnaire (Lakka and Salonen 1992; Lakka et al. 1994). This questionnaire is modified from the Minnesota leisure‐time activity questionnaire. In the KIHD questionnaire, participants are asked to fill in all types of physical activities (e.g., running, skiing, cycling, walking, household work, gardening, snow shoveling) they have performed during the past 12 months. Participants also had an opportunity to report their own activities. In addition, for each of the reported activities, the participants filled in the intensity (0 = recreational, 1 = conditioning, 2 = brisk conditioning, 3 = competitive, strenuous exercise), frequency (times per month), and the mean duration of the activity. We set a metabolic equivalent of task (MET) value for each activity based on the defined intensity for that activity using the previously reported MET databases (Ainsworth et al. 2011). MET describes the absolute intensity of the activity as the ratio between the metabolic rate during the activity and the metabolic rate at rest (1 MET = 3.5 mL O_2_ ∙ kg^−1^ ∙ min^−1^ or 1 kcal ∙ kg^−1^ ∙ h^−1^). After that, we calculated the volume of each activity (METh) as a product of the MET value, average duration, and frequency of the activity. Then, the sum of the volumes of the activities performed was calculated to quantify the total volume of LTPA in hours of metabolic equivalent of task per week (METh/wk).

LTPA was exploited in the analyses both as a continuous and categorical variable. The LTPA was divided into five categories: (1) < 8.3 METh/wk, (2) 8.3–< 16.7 METh/wk, (3) 16.7–< 33.3 METh/wk, (4) 33.3–< 50 METh/wk, and (5) ≥ 50 METh/wk. These categories are based on their comparability to the current physical activity guidelines (World Health Organization 2020): 8.3 METh/wk. (=500 METmin/wk) corresponds to the minimum amount of physical activity in the current guidelines, 16.7 METh/wk (=1000 METmin/wk) is double, 33.3 METh/wk (=2000 METmin/wk) is threefold, and 50 METh/wk (=3000 METmin/wk) is fourfold the minimum amount of physical activity in the current guidelines.

### 2.3 Accelerometer‐Based Physical Activity

We measured physical activity using a triaxial accelerometer ActiGraph wGT3X‐BT (ActiGraph LLC, Pensacola, Florida, USA) in the last clinical examination in 2017–2018. ActiGraphs were initialized using ActiLife software (version 6.13.3; ActiGraph LLC, Pensacola, Florida, USA) to collect data at 30 Hz. Each participant wore the accelerometer either on their nondominant or dominant wrist for 24 h per day over seven consecutive days. We instructed participants to take the accelerometer off only during water‐based activities (e.g., shover, swim, or sauna). In addition to wearing the accelerometer, participants also filled in a diary to report bedtimes, waking up times, times for intentional physical activity/exercise, and naps.

After each measurement, we uploaded the measurement and saved it in raw format (raw data file .gt3x) using ActiLife software (version 6.13.3). We used the R‐package GGIR version 3.00.09 (http://cran.r‐project.org; van Hees et al. 2013) to analyze raw accelerometer data. In GGIR, the signal is processed using autocalibration. In the autocalibration, local gravity is used as a reference, sustained abnormally high values and non‐wear time are detected, and the average magnitude of dynamic acceleration corrected for gravity (Euclidean Norm minus 1 g, ENMO, milligravitational [mg] units) is calculated (van Hees et al. 2014) An epoch length of 5 s was used. Non‐wear time was defined based on the standard deviation (SD) and range of the raw acceleration signals at each axis, and they were calculated per 60‐min windows with a 15‐min sliding window (van Hees et al. 2013). A postcalibration error bigger than 10 mg (van Hees et al. 2014) caused the exclusion of a participant. At least 16 h of wear time was needed to regard a day as valid, and at least four valid days, including at least one weekend day, were needed to regard a measurement as valid and to be included in the analyses. Sleep period time was defined based on the sleep diary kept by the participants during the measurement. When sleep diary information was unavailable, the sleep period time was defined using a previously published algorithm (van Hees et al. 2015).

We analyzed each day of the 7‐day measurement separately. Thus, participants' outcome variables are based on the complete 24‐h cycle (=1440 min) and averaged across all valid days. Using the values of the variables from each day, we calculated the weighted average for each variable across all days, where weekend days are always weighted 2/5 relative to the contribution of weekdays. Each variable was also standardized. For each participant, the following variables were generated: (1) mean acceleration during the waking time (mg), (2) mean acceleration during the sleep period time (mg), (3) intensity gradient during the waking time, (4) time accumulated in inactivity during the waking time (min), (5) time accumulated in light physical activity during the waking time (LIPA, min), and (6) time accumulated in moderate to vigorous physical activity during the waking time (MVPA, min). The variables describing mean acceleration represent the overall movement and physical activity level based on the average acceleration/intensity during a given time period (Rowlands et al. 2018). Intensity gradient reflects the acceleration intensity distribution during the given time period and describes the negative curvilinear relationship between physical activity intensity and time accumulated at that intensity. Intensity gradient was calculated in GGIR using the previously described method. (Rowlands et al. 2018) Finally, we applied previously determined cut points to calculate time accumulated in inactivity (accelerations 0–30 mg), LIPA (accelerations 30–100 mg), and MVPA (accelerations > 100 mg).

### 2.4 ΔmetaboAge and MetaboHealth

We draw venous blood samples from each participant after an overnight fast at each of the three clinical examinations. We used a high‐throughput NMR metabolomics platform for the quantification of metabolomic biomarkers (Nightingale Health Ltd., Helsinki, Finland). The details of the experimentation have been described elsewhere (Soininen et al. 2015; Würtz et al. 2017). The metabolomic biomarkers were then used to construct MetaboAge (van den Akker et al. 2020) and MetaboHealth (Deelen et al. 2019). MetaboAge and MetaboHealth calculations were performed using the R package MiMIR (Bizzarri et al. 2022); the scripts are available on D. Bizzarri's GitHub repository (https://github.com/DanieleBizzarri/MiMIR). MetaboAge and MetaboHealth combine different serum metabolites to predict biological age (van den Akker et al. 2020) and assess mortality risk (Deelen et al. 2019). Both ΔmetaboAge and MetaboHealth provide valuable insights into an individual's metabolic state and potential health risks, offering complementary information to traditional clinical measures.

The MetaboAge algorithm incorporates 63 distinct metabolites to generate an individual's metabolomic age score. The algorithm, trained using elastic net regression based on the association with chronological age in 25,000 individuals, integrates a comprehensive range of metabolic markers. The final MetaboAge score represents an individual's metabolomic age, where higher values indicate a more advanced metabolomic age profile. MetaboAge is expressed as MetaboAge acceleration (ΔmetaboAge), which represents the age‐independent difference between MetaboAge and chronological age at the same time point. ΔmetaboAge is calculated by regressing the difference between MetaboAge and chronological age onto chronological age and using residuals from this regression (van den Akker et al. 2020).

The MetaboHealth algorithm was trained on mortality data from 44,000 individuals and utilizes 14 key metabolites. MetaboHealth is a mortality score that typically ranges between −2 and 3 in most cohorts. Higher MetaboHealth scores indicate increased mortality risk, and a one‐unit increase in MetaboHealth has been associated with a 2.73 times higher mortality risk (Deelen et al. 2019).

### 2.5 Covariates

Covariate data used in this study were collected at baseline in the first clinical examination. We measured participants' height to the nearest 0.1 cm using stadiometers (KaWe) and weight in light indoor clothing to the nearest 0.1 kg using medical scales (SECA alpha 770). We calculated body mass index (BMI, kg/m^2^) by dividing weight in kilograms (kg) by height in meters squared (m^2^). We measured waist circumference (cm) at midway between the lowest rib and the iliac crest using a tape measure (Prym) and defined fat percentage using bioimpedance (Inbody 720, Biospace Co Ltd., Seoul, Korea). We measured systolic and diastolic blood pressure from the right arm in a sitting position using a mercury sphygmomanometer. The mean of two successive blood pressure readings is reported. Blood samples were taken after an overnight fast to define the levels (mmol/L) of total cholesterol, high‐density lipoprotein cholesterol, low‐density lipoprotein cholesterol, and triglycerides. A standardized 2‐h 75 g oral glucose tolerance test (OGTT) was performed to define fasting, 30, and 120 min glucose (mmol/L) and insulin (mU/L) concentrations, and to diagnose possible underlying diabetes. We used the WHO 2006 diagnostic criteria for diabetes (World Health Organization 2006), which define diabetes as fasting plasma glucose ≥ 7.0 mmol/L or 2‐h plasma glucose ≥ 11.1 mmol/L during OGTT. Information on previously diagnosed diabetes was based on self‐report, national registers, or use of diabetes/glucose‐lowering medication.

All participants filled in the questionnaires concerning their health and lifestyle characteristics. The categories for smoking status were (1) never smoked, (2) quit smoking (former smoker), and (3) current smoker. The categories for alcohol consumption were (1) never or quit, (2) less than once a week, and (3) at least once a week. The participants reported their chronic diseases and conditions, including cardiovascular conditions (congestive heart failure, arrhythmias, claudication, angina pectoris, previous heart attack, and stroke), lung diseases (asthma, emphysema, and chronic bronchitis), musculoskeletal disorders (rheumatoid arthritis, osteoporosis), and cancer. Based on the self‐reports, chronic diseases and conditions were categorized as 0 = no chronic diseases or conditions, 1 = one chronic disease or condition, and 2 = two or more chronic diseases or conditions. The participants reported their medications, which were categorized as 0 = no medications, 1 = 1 medication, 2 = 2 medications, 3 = 3 medications, and 4 = 4 or more medications. The highest achieved socioeconomic status for each participant was determined based on the information from Statistics Finland and based on the original classification system (Statistics Finland 1989) and was categorized as high official, low official, self‐employed, and manual workers.

### 2.6 Statistical Analyses

The baseline characteristics of the participants are presented as numbers with percentages for categorical data and means with SD for continuous variables. For the cross‐sectional data at baseline, we applied multiple regression analyses with bootstrapped‐style 95% confidence intervals (CIs) with 10,000 replications and with linear and quadratic terms for LTPA to analyze the association between the continuous LTPA and ΔmetaboAge and MetaboHealth. Quadratic models were reported if the quadratic term was found to be significant.

For the longitudinal data, linear mixed models with a random intercept and random slope were applied to investigate the association between the baseline categorical LTPA and the standardized residual change in ΔmetaboAge and MetaboHealth from baseline to the 5‐year and to the 16‐year follow‐up time points. For sensitivity purposes, we also analyzed linear mixed models only among individuals who did not have diabetes at baseline. This was done since individuals who had diabetes at the first clinical examination were excluded from the second clinical examination. In addition to this, we compared baseline MetaboAge among those who did and did not participate in both the 5‐year and the 16‐year follow‐up. We applied linear regression analyses with bootstrapped style 95% CIs with 10,000 replications to investigate whether the increasing gap between MetaboAge and the chronological age during the follow‐ups was caused by survival bias. The standardized residual change score was used to calculate the change from baseline to 5 years and from baseline to 16 years due to the strong negative correlation between the baseline value and the change in the outcomes (ΔmetaboAge and MetaboHealth) from baseline to follow‐up. To calculate the residual change score, we first performed a linear regression model where the baseline value of the outcome was set as an independent variable to predict the follow‐up value of the outcome. The predicted values from the model were subtracted from the corresponding measured follow‐up values to get the residual change score. Finally, the residual change score was standardized for the analyses. The residual change scores were standardized to improve comparability. Standardization was performed by dividing each residual by the SD of the residuals, resulting in *Z*‐scores with a mean of 0 and a SD of 1.

We also analyzed the association between the standardized residual change in LTPA and the standardized residual change in the outcomes from baseline to the 16‐year follow‐up using general linear models with bootstrapped style 95% CIs with 10,000 replications. For these analyses, the standardized residual change in LTPA was categorized into three groups to indicate a decreased (the standardized residual change in LTPA ≤ −0.5 SD), stable (the standardized residual change in LTPA = −0.5–0.5 SD) or increased (the standardized residual change in LTPA ≥ 0.5 SD) volume of LTPA. This approach was chosen based on its robustness, as it uses two time points to calculate the change and considers possible errors in the measurements by using ±0.5 SD as limits for the categories.

For all the above‐mentioned analyses, we developed a crude model that was adjusted for sex and age and the fully adjusted model that was adjusted for sex, age, chronic diseases, smoking, alcohol consumption, socioeconomic status, follow‐up time (only in the short‐term and long‐term models), BMI, and medications. *p*‐values < 0.05 were considered statistically significant. To address the issue of multiple comparisons between different LTPA categories and to control the family‐wise error rate, the Bonferroni correction was applied. The adjustment was applied to post hoc comparisons to ensure robust conclusions. The correction involved multiplying the nominal *p*‐value by four, and all *p*‐values that remained lower than the 0.05 threshold were considered to be statistically significant. The Bonferroni correction was made by using the mcompare (Bonferroni) option in Stata.

For the cross‐sectional accelerometer‐based physical activity data at the last clinical examination, a linear regression analysis (95% CIs) was applied to investigate the associations between the different accelerometer‐based physical activity variables and ΔmetaboAge and MetaboHealth. The crude model was adjusted for sex and age, and the fully adjusted model was adjusted for sex, age, body mass index, chronic diseases, smoking, alcohol consumption, and net income. *p*‐values ≤ 0.001 were considered statistically significant. The Bonferroni‐Holm correction was applied to control the family‐wise error rate. The Bonferroni‐Holm correction is a stepwise approach that adjusts for the number of tests while maintaining greater statistical power than the traditional Bonferroni correction, ensuring that the conclusions drawn are more robust and reliable.

All analyses were performed with Stata/MP 18.0 (StataCorp, TX, USA).

## 3 Results

### 3.1 Participants' Characteristics

The characteristics of the study participants (*n* = 1816) are described in Table 1. At baseline, the mean age of the participants was 61.6 years (SD = 2.9) and 53% were women. At the second clinical examination, the mean age of the participants was 66.4 years (SD = 2.8) and 54% were women. At the last clinical examination, the mean age of the participants was 76.9 years (SD = 2.7) and 55% were women. The mean short‐term follow‐up time was 4.9 years (SD = 0.8), and the mean long‐term follow‐up time was 15.8 years (SD = 0.8).

**TABLE 1:** Participants' characteristics.

Characteristics	*n*	
Sex		
Women, *n* (%)	1816	971 (53)
Age, years, mean (SD)	1816	61.6 (2.9)
Socioeconomic status, *n* (%)		
High official	1816	262 (14)
Low official		779 (43)
Self‐employed		164 (9)
Labourers		611 (34)
Body mass index, kg/m^2^, mean (SD)	1816	27.6 (4.5)
Waist circumference, cm, mean (SD)	1814	95.4 (12.9)
Women	970	91.0 (12.7)
Men	844	100.5 (11.2)
Fat percentage, %, mean (SD)	1737	29.1 (8.2)
Women	930	33.9 (6.9)
Men	807	23.6 (5.9)
Smoking, *n* (%)		
Never	1816	759 (42)
Former		634 (35)
Current		423 (23)
Chronic diseases, *n* (%)		
None	1816	711 (39)
1 disease		649 (36)
≥ 2 diseases		456 (25)
Alcohol use, *n* (%)		
Never/quit	1816	130 (7)
< 1 per week		754 (42)
Weekly		932 (51)
Medication, *n* (%)		
None	1816	799 (44)
1 medication		310 (17)
2 medications		239 (13)
3 medications		180 (10)
≥ 4 medications		288 (16)
Glucose, mmol/L, mean (SD)		
Fasting glucose	1816	5.8 (1.3)
30 min glucose	1769	9.5 (2.2)
120 min glucose	1773	7.8 (3.3)
Insulin, mU/L, mean (SD)		
Fasting insulin	1816	10.8 (11.8)
30 min insulin	1771	71 (47.8)
120 min insulin	1774	75.3 (63.4)
Total cholesterol, mmol/L, mean (SD)	1815	5.93 (1.1)
Triglycerides, mmol/L, mean (SD)	1815	1.5 (0.8)
HDL‐cholesterol, mmol/L, mean (SD)	1815	1.6 (0.4)
LDL‐cholesterol, mmol/L, mean (SD)	1785	3.65 (0.9)
Diabetes status at baseline, yes *n* (%)	1814	267 (14.7)
LTPA, METh/week, mean (SD)	1816	46.3 (40.4)
Baseline		
MetaboAge, years, mean (SD)	1816	59.1 (7.8)
ΔmetaboAge, years, mean (SD)	1816	0.012 (7.8)
MetaboHealth, units, mean (SD)	1816	−0.016 (0.6)
Short‐term follow‐up		
MetaboAge, years, mean (SD)	987	55.4 (9.3)
ΔmetaboAge, years, mean (SD)	987	−0.2 (9.2)
MetaboHealth, units, mean (SD)	987	−0.004 (0.5)
Long‐term follow‐up		
MetaboAge, years, mean (SD)	744	63.9 (7.3)
ΔmetaboAge, years, mean (SD)	744	−0.2 (7.2)
MetaboHealth, units, mean (SD)	744	−0.019 (0.5)

Abbreviations: HDL, high‐density lipoprotein; LDL, low‐density lipoprotein; LTPA, leisure‐time physical activity; SD, standard deviation.

### 3.2 LTPA and Baseline ΔmetaboAge and MetaboHealth

At baseline, a greater volume of LTPA was non‐linearly associated with a lower ΔmetaboAge in the crude (*p* = 0.0498) and in the fully adjusted model (*p* = 0.047) and with a lower MetaboHealth score in the crude (*p* < 0.001) and in the fully adjusted model (*p* < 0.001; Table 2).

**TABLE 2:** The association between the volume of leisure‐time physical activity (LTPA) and ΔmetaboAge and MetaboHealth in late midlife (baseline). Only complete cases were included in the analyses.

Dependent variable	Independent variable	Crude model			Fully adjusted model	
		*n*	*b* (95% CI)	*p*	*b* (95% CI)	*p*
Baseline						
ΔmetaboAge	LTPA	1816	−0.024 (−0.044 to −0.004)	0.0498	−0.02 (−0.04 to −0.005)	0.047
	LTPA^2^	1816	0.0001 (0.00003–0.0002)		0.0001 (0.00001–0.0002)	
MetaboHealth	LTPA	1816	−0.004 (−0.005 to −0.002)	< 0.001	−0.003 (−0.004 to −0.002)	< 0.001
	LTPA^2^	1816	0.00001 (0.00001–0.00002)		0.00001 (0.000004–0.00002)	

*Note:* Crude model: adjusted for sex and age. Fully adjusted model: adjusted for sex, age, chronic diseases, smoking, alcohol consumption, socioeconomic status, follow‐up time (only in short‐term and long‐term models), body mass index, and medication.

### 3.3 LTPA and Change in ΔmetaboAge

**FIGURE 2:**
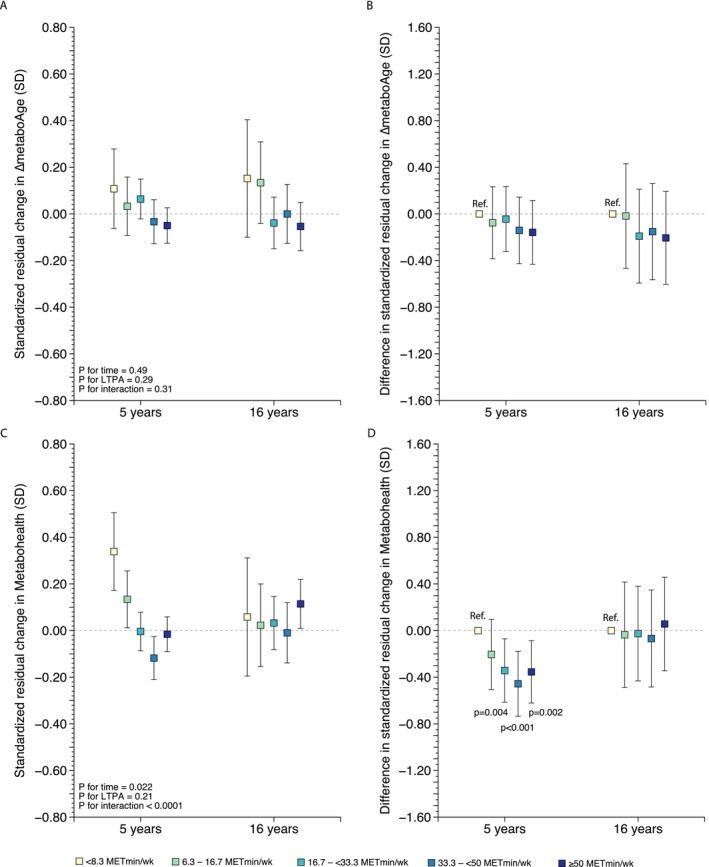
The association between the volume of leisure‐time physical activity (LTPA) in late midlife and the standardized residual change in (A) ΔmetaboAge and (C) MetaboHealth and the mean difference between the LTPA categories in the standardized residual change in (B) ΔmetaboAge and (D) MetaboHealth in the 5‐year and 16‐year follow‐up. Analyses were adjusted for sex, age, chronic diseases, smoking, alcohol consumption, socioeconomic status, follow‐up time, body mass index, and medication. Analyses in (B) and (D) were Bonferroni adjusted for multiple comparisons. Range plots with capped spikes indicate 95% confidence intervals.

There was no significant interaction between time and LTPA on the standardized residual change in ΔmetaboAge neither in the crude (*p* = 0.32; Figure S1A) nor in the fully adjusted model (*p* = 0.31; Figure 2A). The volume of LTPA was not associated with the change in ΔmetaboAge neither in the 5‐year follow‐up nor in the 16‐year follow‐up (Figure 2A,B).

### 3.4 LTPA and Change in MetaboHealth

We found a significant interaction between time and LTPA on the standardized residual change in MetaboHealth score in the crude (*p* < 0.0001) (Figure S1C) and in the fully adjusted model (*p* < 0.0001) (Figure 2C). In the 5‐year follow‐up, belonging to the fourth LTPA category was associated with a decreased MetaboHealth score (*p* < 0.001; Figure 2D). Furthermore, belonging to the third LTPA category (*p* = 0.004) and to the fifth LTPA category (*p* = 0.002) was associated with a stable MetaboHealth score in the 5‐year follow‐up. The MetaboHealth score in these three LTPA categories was significantly different from the first LTPA category in which an increased MetaboHealth score was detected. In the 16‐year follow‐up, there was no association between the volume of LTPA and the change in MetaboHealth score (Figure 2C,D).

### 3.5 Long‐Term Change in LTPA, ΔmetaboAge, and MetaboHealth

**FIGURE 3:**
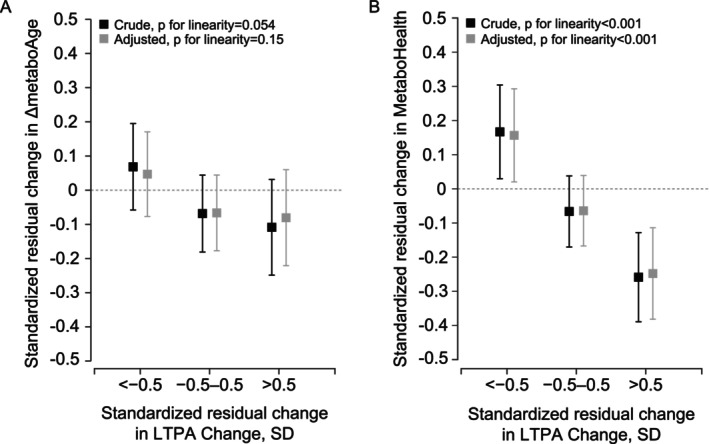
The association between the standardized residual change in leisure‐time physical activity (LTPA) and the standardized residual change in (A) ΔmetaboAge and (B) MetaboHealth during the 16‐year follow‐up (*n* = 706). Analyses in the crude model were adjusted for sex and age, and in the fully adjusted model for sex, age, chronic diseases, smoking, alcohol consumption, socioeconomic status, follow‐up, body mass index, and medication.

Figure 3 describes the standardized residual change in LTPA (METh) in relation to the standardized residual change in ΔmetaboAge (A) and MetaboHealth score (B) during the 16‐year follow‐up. An increase in the volume of LTPA from late midlife to old age was associated with a decreased MetaboHealth score (i.e., lower mortality risk) while at the same time, a decrease in the volume of LTPA was associated with an increased MetaboHealth score in both models (*p* < 0.001). There was no association between the long‐term change in the volume of LTPA and the long‐term change in ΔmetaboAge neither in the crude (*p* = 0.054) nor in the fully adjusted model (*p* = 0.15).

### 3.6 Accelerometer‐Based Physical Activity and ΔmetaboAge and MetaboHealth

We had accelerometer‐based physical activity data from 706 participants at the last clinical examination. We found that mean acceleration during waking time, intensity gradient, and MVPA were associated with a lower MetaboHealth score in both models, and time accumulated in inactivity was associated with a higher MetaboHealth score only in the crude model (Table S1). No associations were found between mean acceleration during sleep period time and LIPA and MetaboHealth in either of the models. None of the accelerometer‐based physical activity variables were found to be associated with ΔmetaboAge (Table S2).

### 3.7 Sensitivity Analyses

For sensitivity analyses, we also analyzed the volume of LTPA and the 5‐year and the 16‐year standardized residual change in ΔmetaboAge and MetaboHealth score among participants who did not have diabetes at baseline (*n* = 1547). As described in Table S3, the results remained similar in the sensitivity analyses. The volume of LTPA was associated with the standardized residual change in MetaboHealth score in the 5‐year but not in the 16‐year follow‐up. There was no association between the volume of LTPA and the standardized residual change in ΔmetaboAge either in the 5‐year or in the 16‐year follow‐up.

We detected an increase in the gap between MetaboAge and the chronological age during the long term but not during the short‐term follow‐up. The baseline MetaboAge was 0.71 years higher (95% CI −0.03 to 1.45, *p* = 0.06) among those who did not participate in the 16‐year follow‐up measurements compared to those who participated in the 16‐year follow‐up measurements. The baseline MetaboAge did not differ (0.01 years, 95% CI −0.71 to 0.73, *p* = 0.98) between those who did and did not participate in the 5‐year follow‐up measurements.

## 4 Discussion

In this prospective cohort study, our most prominent findings were that changes in the volume of LTPA from late midlife to old age are reflected in the MetaboHealth score, suggesting that increasing volumes imply decelerated metabolic aging and decreasing volumes imply accelerated metabolic aging in the 16‐year follow‐up. Furthermore, a greater volume of LTPA in late midlife was associated with a lower metabolomics‐based biological age in the 5‐year follow‐up when the volume of LTPA was at least twice the current minimum recommendation of physical activity, and that LTPA was more strongly associated with the MetaboHealth score than MetaboAge. The results based on the cross‐sectional device‐based physical activity measurements are consistent with the findings obtained from the self‐reported physical activity, also supporting the inverse relationship of physical activity with MetaboHealth but not with ΔmetaboAge. Overall, our findings suggest that physical activity over the transition years from late midlife to old age can protect against metabolic aging. The results support the importance of physical activity for biological aging in later life.

Our findings suggest that increasing one's volume of LTPA from late midlife to old age can decrease MetaboHealth‐based biological age, and thus increasing LTPA might help to decelerate biological aging. It was also noteworthy that a decrease in the volume of LTPA during 16 years of follow‐up was associated with an increased MetaboHealth‐based biological age implying accelerated biological aging. Even though longitudinal changes in metabolomics‐based aging markers are not well‐studied, one study reported based on a 10‐year retrospective analysis that becoming more physically active by starting to exercise according to the physical activity guidelines was associated with a lower MetaboHealth score (Kuiper et al. 2024). In addition, there are studies which have reported comparable findings in more general terms supporting a favorable role of increased physical activity for biological aging in late midlife such as being free from major chronic diseases and having good physical, cognitive, and mental health (Shi et al. 2024). Taken together, it seems that by adopting a more active lifestyle and increasing the volume of LTPA even relatively late in life can lead to measurable and significant long‐term health and metabolic benefits and in that way promote healthier aging. And vice versa, diminishing amounts of physical activity with age might lead to accelerated biological aging and an increase in one's mortality risk.

We observed that LTPA in late midlife was associated with the change in MetaboHealth after the 5‐year but not after the 16‐year follow‐up. A greater volume of LTPA in late midlife resulted in a lower MetaboHealth‐based biological age at the 5‐year follow‐up when the volume of LTPA in late midlife was high enough. Based on our results, reaching the minimum recommended level of physical activity (8.3 METh/wk) in the current guidelines (World Health Organization 2020) in late midlife was not enough to slow down metabolic aging. Instead, at least twice as much physical activity as the minimum recommended amount of physical activity might be required to delay metabolic aging. In fact, accumulating LTPA on average 33.3–< 50 METh/wk was associated with a decrease in MetaboHealth, whereas LTPA of more than 50 METh/wk and 16.7–33.3 METh/wk resulted in a stable MetaboHealth in 5 years. The required amount of physical activity to delay metabolic aging in our study is higher than in another study with a 10‐year longitudinal follow‐up, where adherence to the physical activity guidelines, meaning the minimum recommended level of physical activity, was enough to be associated with a lower MetaboHealth score (Kuiper et al. 2024). The discrepancy between the studies might be explained by the assessment of the volume of physical activity and the differences in the length of the follow‐up periods. Collectively, these findings imply that regular physical activity most days of the week seems to be required to maximize LTPA's potential to decelerate metabolic aging, at least in a 5–10‐year perspective.

The observed lack of associations of LTPA with MetaboHealth and ΔmetaboAge at the 16‐year follow‐up might depend on several factors. First, survival bias might have introduced selection bias, resulting in that those who remained in the study might differ systematically from those who dropped out. Second, the changes or fluctuations in LTPA over such a long‐term time period might have affected the beneficial effects of earlier physical activity that may not persist indefinitely if LTPA is not maintained or increased over time. Third, the performance of metabolomics‐based aging biomarkers over the long term is not yet fully established. Their stability and reliability may evolve due to the interplay of biological, lifestyle, environmental, and methodological factors over 16 years, potentially introducing variability in biomarkers over time. For example, participants might have experienced significant life and health events (including onset of diseases such as diabetes or cardiovascular disease) and changes in other health‐related behaviors, potentially confounding the relationship in the long term. We were able to consider a wide range of potential confounders, but we cannot exclude the possibility of potential unmeasured covariates or emerging confounding factors diminishing the effect of baseline LTPA on the aging biomarkers. In addition, biomarkers calibrated to midlife profiles may lose their ability to accurately reflect biological age or predict outcomes in older individuals due to changing metabolic dynamics. However, in a recent study, the authors concluded that the stability of metabolomics‐based aging biomarkers over time seems to be relatively stable (Kuiper et al. 2024) indicating their suitability for follow‐ups of different lengths, even though more thorough examinations are needed.

Only a few studies have investigated the cross‐sectional association between physical activity and metabolomics‐based markers of biological aging. Two studies align with our findings and have linked higher physical activity levels or the adherence to physical activity guidelines to more favorable metabolomic profiles and a lower metabolomics‐based biological age (Hertel et al. 2016; Kuiper et al. 2024). Three other studies do not support our finding on the association of LTPA with MetaboAge and MetaboHealth in late midlife in the cross‐sectional analyses. In the UK Airwave cohort including 19–65‐year‐old adults (mean age 41 years), the metabolomic age based on urine and serum metabolomics was not associated with physical activity (Robinson et al. 2020). In the NESDA study, physical activity was neither associated with metabolomic aging measured as MetaboAge in adults at the mean age of 42 years (Jansen et al. 2021). Likewise, in the Doetinchem Cohort study, physical activity was not included in the top‐ranked predictors of metabolomic aging that was measured as MetaboHealth in adults aged 36–75 years (mean age 55 years) when considering other possible exposures (Smit et al. 2024). The inconsistent findings in these studies compared to our study might be at least partly explained by the younger study populations and the differences in biosamples, methodology, or the calculation of the metabolomics‐based biological age score.

The association between LTPA and metabolomics‐based biological age might be explained by the fact that physical activity has the potential to affect at the cellular and molecular level by mitigating the age‐associated changes in the hallmarks of aging, including mitochondrial function and nutrient sensing mechanisms (Rebelo‐Marques et al. 2018) that are closely coupled with metabolism and metabolic aging (López‐Otín et al. 2023). Our findings align with studies investigating the association of physical activity and/or physical fitness with other molecular aging biomarkers such as epigenetic and transcriptomic aging (Voisin et al. 2024) and cellular senescence biomarkers (Englund et al. 2021). Another possible explanation might concern the association of physical activity and the metabolites involved in MetaboAge and MetaboHealth. This is supported by the known associations of physical activity with the metabolome in general (Kelly et al. 2020) and in particular with the single metabolites found in MetaboAge and MetaboHealth that are involved in lipoprotein and fatty acid metabolism, glycolysis, fluid balance, and inflammation (Deelen et al. 2019; van den Akker et al. 2020). In addition, physically active people have been shown to differ from their more inactive counterparts in their metabolome regarding the ratio of polyunsaturated fatty acids to total fatty acids and levels of glucose and isoleucine, all of which are found in MetaboAge and MetaboHealth (Kujala et al. 2013). This suggests that these differences might also be reflected in metabolomics‐based markers of biological aging. However, more studies are needed to study the exact mechanisms behind the association between LTPA and metabolomics‐based biological age.

Both self‐reported and device‐based physical activity were found to be more strongly associated with MetaboHealth than ΔmetaboAge, supporting findings from previous studies (Kuiper et al. 2023, 2024) suggesting that MetaboHealth is superior to MetaboAge in reflecting biological age and predicting age‐related health outcomes and mortality risk. Similarly, MetaboAge has been shown to be less responsive to longitudinal lifestyle modification and associated with fewer lifestyle factors (Kuiper et al. 2024) and classical health biomarkers (Bogaards et al. 2022) than MetaboHealth. The discrepancy between MetaboAge and MetaboHealth might be explained by the fact that MetaboAge is trained on chronological age and MetaboHealth is trained on mortality. MetaboAge increases with time and describes how old the body appears biologically compared to the chronological age (van den Akker et al. 2020), as MetaboHealth is linked to health outcomes related to aging and longevity, specifically the risk of mortality (Deelen et al. 2019). Aging biomarkers trained on longitudinal mortality or other biophysiological information have been shown to outplay age‐trained biomarkers when predicting mortality and reflecting biological age measures such as frailty due to their ability to capture more biologically relevant aging signals (Kuiper et al. 2023; Rutledge et al. 2022). Other suggested reasons are the selection bias of not including fast‐agers who usually die sooner when modeling aging biomarkers based on age and the fact that metabolic aging based on cross‐sectional data might not link to longitudinal metabolic changes (Nelson et al. 2020). Taken together, our study and previous studies support the use of aging biomarkers trained on longitudinal physiologically relevant information, and that physical activity is more closely linked to age‐related health outcomes and mortality risk, which are better portrayed by MetaboHealth than ΔmetaboAge.

The large gap between the mean values for MetaboAge and chronological age found in our study is in line with a recent study (Kuiper et al. 2024). In both studies, the gap increased with longer follow‐up years. Survival bias in our study is one potential reason for this, as the sample at baseline might differ from the sample at the 5‐year and 16‐year follow‐up. The baseline MetaboAge in those who did not participate in the follow‐ups was slightly but not statistically significantly higher than in those who were able to participate in the last clinical examinations, meaning that the second and third samples might represent more metabolically healthy people. In addition, it is yet not known how age‐trained metabolomics‐based aging biomarkers suit older populations, especially if they have been validated using younger cohorts (Nelson et al. 2020) when people dying at younger ages are not included in the development and validation of the model. All this suggests that the suitability of MetaboAge in old age should be investigated further.

This study has several strengths, including a well‐characterized birth cohort, a large sample size, and both a short‐ (5‐year) and long‐term (16‐year) follow‐up. We were able to measure the total volume of LTPA in the same way both at baseline and at the long‐term follow‐up using the validated KIHD questionnaire. We were also able to validate the results using cross‐sectional accelerometry data from the last clinical examination. We used MetaboAge and MetaboHealth, which are composite measures, trained and validated on large cohort data samples, and easily determined from fasting blood samples that is a minimally invasive and reliable measurement containing little technical variability. The analyses were also adjusted for multiple confounders.

Self‐report methods for physical activity assessment are widely used and stand out from the other methods for their practicality and low cost and burden when assessing the physical activity of a large number of individuals and of longer follow‐ups (Prince et al. 2008). However, they are prone to recall and information biases due to overreporting and/or misreporting, and we acknowledge that the KIHD questionnaire used in our study might potentially suffer from these limitations. To address that, we studied the association of physical activity with MetaboAge and MetaboHealth in old age in a cross‐sectional setting using device‐based physical activity data. These results validate our findings based on self‐reported physical activity data showing that physical activity is inversely related to MetaboHealth but not to ΔmetaboAge. More specifically, these results suggest that the intensity of physical activity and time spent at higher intensities seem to be important for metabolic aging. Taken together, the findings based on both LTPA and accelerometer‐based physical activity data support the importance of a physically active lifestyle, possibly emphasizing regular daily periods of higher intensity, for metabolomics‐based biological aging in later life. However, in the future, these cross‐sectional findings should be validated using device‐based measurements of physical activity in longitudinal settings.

When interpreting the results, the following limitations should also be addressed. First, the follow‐up data in old age are susceptible to survival bias due to the increasing morbidity and mortality rates that hinder participation in clinical measurements. This attrition over the follow‐up period might have resulted in a healthier and/or more active study population the longer the follow‐up, potentially biasing the results and limiting their generalizability. Furthermore, only individuals who did not have diabetes at the first clinical visit were invited to the second clinical examination. However, according to the sensitivity analyses (Table S3) this did not affect the results. Second, due to the lack of physical activity data at the 5‐year follow‐up in 2007–2008, we were not able to assess the possible short‐term changes in physical activity that might have been relevant in this aging study population. Third, as the data originate from individuals born between 1934 and 1944 in the Helsinki region, Finland, the regional and temporal specificity of the cohort may limit their direct applicability to other populations and age groups. Our study population consisted of community‐dwelling Caucasians, limiting the generalizability of the findings to other ethnic groups. As it was voluntary to attend child welfare clinics, our subjects may not represent the population born in Helsinki even though the attendance was free. In addition, our subjects were children during the Second World War and may have suffered from food shortages, potentially influencing the applicability of the study findings to contemporary cohorts. Nonetheless, the results are likely relevant across various populations, though the magnitude of their effects may differ. However, additional studies in more diverse settings are needed to confirm these results, and future research should prioritize diverse and global cohorts to enhance the external validity of these findings. Finally, due to the non‐experimental study design, no inferences on causality can be made.

In conclusion, our findings suggest that a greater volume of LTPA in late midlife is associated with lower ΔmetaboAge and MetaboHealth in late midlife and with a lower MetaboHealth after the 5‐year follow‐up, especially when the volume of LTPA is high enough (≥ 16.7 METh/wk). In addition, increasing one's volume of LTPA from late midlife to old age might protect against metabolic aging during a 16‐year follow‐up, and vice versa; decreasing physical activity might accelerate biological aging in the corresponding time period. The found associations were stronger for MetaboHealth than MetaboAge. These findings, based on self‐reported physical activity, align with the cross‐sectional findings from the device‐based physical activity measurements, supporting the found inverse association of physical activity with MetaboHealth but not with ΔmetaboAge. Our findings indicate that both an increase and a decrease in physical activity levels with age, even later in life, may influence biological aging, and that physical activity appears to be more strongly associated with MetaboHealth, which better than MetaboAge represents age‐related health outcomes and mortality risk. Collectively, the study and its findings provide a novel perspective on the relationship between physical activity and metabolomics‐based biological aging and support the importance of, and increase the understanding of, the role of physical activity in biological aging. Future research could expand to study the relationship between physical activity and metabolomics‐based aging markers using device‐based physical activity data in longitudinal and interventional study designs, as well as looking into the possible mechanisms and the possible causality behind this relationship.

## Author Contributions

N.S.W., J.G.E., K.R., T.M.M., and M.K.L. have contributed to the conception and design of the study. K.N. has been responsible for the analysis and calculation of the metabolomics‐based aging markers. N.S.W. conducted the statistical analyses. K.R. and N.S.W. contributed to the analysis and interpretation of data. K.R. wrote an initial draft of the manuscript, and all authors provided critical feedback and helped shape the research, analysis, and manuscript.

## Conflicts of Interest

The authors declare no conflicts of interest.

## Supporting information


**Figure S1.** The association between the volume of leisure‐time physical activity (LTPA) in late midlife and the standardized residual change in (A) ΔmetaboAge and (C) MetaboHealth and the mean difference between the LTPA categories in the standardized residual change in (B) ΔmetaboAge and (D) MetaboHealth in the 5‐year and the 16‐year follow‐up. Analyses were adjusted for sex and age. Analyses in B and D were Bonferroni adjusted for multiple comparison. Range plots with capped spikes indicate 95% confidence intervals.

**Table S1.** The association between different accelerometer‐based physical activity variables and MetaboHealth in old age (the third clinical examination in 2017–2018).
**Table S2**. The association between different accelerometer‐based physical activity variables and ΔmetaboAge in old age (the third clinical examination in 2017–2018).
**Table S3**. Sensitivity analyses on participants who did not have diabetes at the first clinical visit (baseline).

## Data Availability

The data that support the findings of this study are available upon reasonable request.
