# Supplementary figures and images for: Associations Between Leisure‐Time Physical Activity and Metabolomics‐Based Markers of Biological Aging in Late Midlife: Short‐Term and Long‐Term Follow‐Up
Source: Aging Cell. 2025 Mar 10;24(6):e70033. doi: 10.1111/acel.70033 (PMC12151911; doi:10.1111/acel.70033)

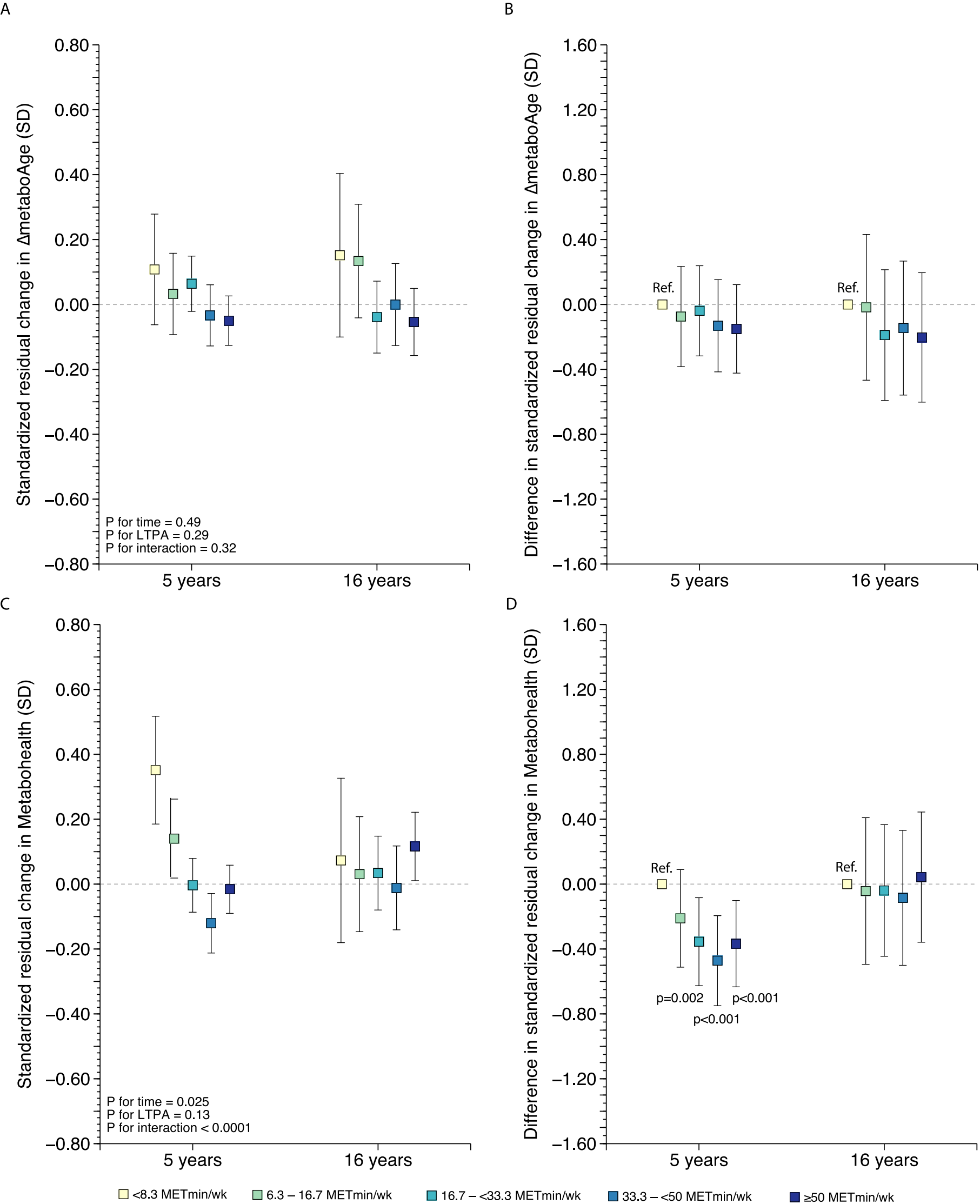

Supplement: Supplementary file 1 — Figure S1. The association between the volume of leisure‐time physical activity (LTPA) in late midlife and the standardized residual change in (A) ΔmetaboAge and (C) MetaboHealth and the mean difference between the LTPA categories in the standardized residual change in (B) ΔmetaboAge and (D) MetaboHealth in the 5‐year and the 16‐year follow‐up. Analyses were adjusted for sex and age. Analyses in B and D were Bonferroni adjusted for multiple comparison. Range plots with capped spikes indicate 95% confidence intervals. [file ACEL-24-e70033-s001.pdf]
