# Supplementary material for: Associations Between Leisure‐Time Physical Activity and Metabolomics‐Based Markers of Biological Aging in Late Midlife: Short‐Term and Long‐Term Follow‐Up
Source: Aging Cell. 2025 Mar 10;24(6):e70033. doi: 10.1111/acel.70033 (PMC12151911; doi:10.1111/acel.70033)
Supplement: Supplementary file 2 — Table S1. The association between different accelerometer‐based physical activity variables and MetaboHealth in old age (the third clinical examination in 2017–2018). Table S2. The association between different accelerometer‐based physical activity variables and ΔmetaboAge in old age (the third clinical examination in 2017–2018). Table S3. Sensitivity analyses on participants who did not have diabetes at the first clinical visit (baseline). [file ACEL-24-e70033-s002.docx]

**Supplementary table 1.** The association between different accelerometer-based physical activity variables and MetaboHealth in old age (the third clinical examination in 2018).

|  | **Crude model (n=706)** | | | |  | **Adjusted model (n=706)** | | | |
| --- | --- | --- | --- | --- | --- | --- | --- | --- | --- |
| **Variable (unit)** | **b** | **95% CI** | **p** |  |  | **b** | **95% CI** | **p** |  |
| Mean acceleration during the waking time (mg) | -0.09 | (-0.13 – -0.06) | <0.001 |  |  | -0.07 | (-0.11 – -0.03) | <0.001 |  |
| Mean acceleration during the sleep period time (mg) | 0.05 | (0.01 – 0.09) | 0.009 |  |  | 0.03 | (0 – 0.07) | 0.07 |  |
| Intensity gradient | -0.09 | (-0.13 – -0.05) | <0.001 |  |  | -0.07 | (-0.12 – -0.03) | 0.001 |  |
| Inactivity (min/day) | 0.07 | (0.03 – 0.1) | 0.001 |  |  | 0.04 | (0 – 0.08) | 0.033 |  |
| Light physical activity (min/day) | -0.05 | (-0.09 – -0.02) | 0.002 |  |  | -0.03 | (-0.07 – 0) | 0.085 |  |
| Moderate to vigorous physical activity (min/day) | -0.09 | (-0.12 – -0.06) | <0.001 |  |  | -0.07 | (-0.11 – -0.04) | <0.001 |  |

*Crude model: adjusted for sex and age.*

*Fully adjusted model: adjusted for sex, age, body mass index, chronic diseases, smoking, alcohol consumption, and net income.*

*The Holm-Bonferroni correction was applied.*

**Supplementary table 2.** The association between different accelerometer-based physical activity variables and ΔmetaboAge in old age (the third clinical examination in 2018).

|  | **Crude model (n=706)** | | | |  | **Adjusted model (n=706)** | | | |
| --- | --- | --- | --- | --- | --- | --- | --- | --- | --- |
| **Variable (units)** | **b** | **95% CI** | **p** |  |  | **b** | **95% CI** | **p** |  |
| Mean acceleration during the waking time (mg) | -0.5 | (-1.05 – 0.07) | 0.081 |  |  | -0.01 | (-0.59 – 0.63) | 0.978 |  |
| Mean acceleration during the sleep period time (mg) | 0.59 | (0.06 – 1.24) | 0.045 |  |  | 0.37 | (-0.13 – 0.97) | 0.181 |  |
| Intensity gradient | -0.08 | (-0.7 – 0.57) | 0.799 |  |  | 0.35 | (-0.27 – 1.01) | 0.283 |  |
| Inactivity (min/day) | 0.31 | (-0.29 – 0.89) | 0.308 |  |  | -0.11 | (-0.72 – 0.47) | 0.715 |  |
| Light physical activity (min/day) | -0.38 | (-0.89 – 0.13) | 0.142 |  |  | -0.01 | (-0.54 – 0.5) | 0.971 |  |
| Moderate to vigorous physical activity (min/day) | -0.54 | (-1.07 – -0.01) | 0.047 |  |  | -0.14 | (-0.69 – 0.43) | 0.618 |  |

*Crude model: adjusted for sex and age.*

*Fully adjusted model: adjusted for sex, age, body mass index, chronic diseases, smoking, alcohol consumption, and net income.*

*The Holm-Bonferroni correction was applied.*

**Supplementary table 3.** Sensitivity analyses on participants who did not have diabetes at the first clinical visit (baseline).

|  |  |  | **Short-term follow-up (5 years)** | | |  | **Long-term follow-up (16 years)** | | |  |  |  |
| --- | --- | --- | --- | --- | --- | --- | --- | --- | --- | --- | --- | --- |
| **Comparison of LTPA categories (METh/wk)** | | | **Mean estimated difference (95% CI), SD** | | **Bonferroni corrected p for pairwise comparison** |  | **Mean estimated difference (95% CI), SD** | | **Bonferroni corrected p for pairwise comparison** | **P for main effect of time** | **P for main effect of LTPA** | **P for time x LTPA Interaction** |
| **MetaboHealth** |  |  |  |  |  |  |  |  |  |  |  |  |
| Crude model | | |  |  |  |  |  |  |  |  |  |  |
| 8.3 – <16.7 | vs | <8.3 | -0.28 | (-0.6 – 0.04) | 0.17 |  | -0.14 | (-0.64 – 0.35) | 1.00 | 0.0642 | 0.124 | <0.0001 |
| 16.7 – <33.3 | vs | <8.3 | -0.42 | (-0.71 – -0.13) | 0.0004 |  | -0.08 | (-0.53 – 0.37) | 1.00 |  |  |  |
| 33.3 – <50 | vs | <8.3 | -0.51 | (-0.81 – -0.22) | <0.0001 |  | -0.14 | (-0.6 – 0.32) | 1.00 |  |  |  |
| ≥50 | vs | <8.3 | -0.43 | (-0.71 – -0.14) | 0.0002 |  | 0 | (-0.44 – 0.44) | 1.00 |  |  |  |
|  |  |  |  |  |  |  |  |  |  |  |  |  |
| Fully adjusted model | | |  |  |  |  |  |  |  |  |  |  |
| 8.3 – <16.7 | vs | <8.3 | -0.27 | (-0.59 – 0.05) | 0.21 |  | -0.06 | (-0.51 – 0.38) | 1.00 | 0.0608 | 0.1981 | <0.0001 |
| 16.7 – <33.3 | vs | <8.3 | -0.4 | (-0.69 – -0.11) | 0.001 |  | -0.13 | (-0.63 – 0.36) | 1.00 |  |  |  |
| 33.3 – <50 | vs | <8.3 | -0.49 | (-0.79 – -0.2) | <0.0001 |  | -0.12 | (-0.58 – 0.34) | 1.00 |  |  |  |
| ≥50 | vs | <8.3 | -0.41 | (-0.69 – -0.12) | 0.001 |  | 0.01 | (-0.43 – 0.46) | 1.00 |  |  |  |
|  |  |  |  |  |  |  |  |  |  |  |  |  |
| **ΔmetaboAge** | |  |  |  |  |  |  |  |  |  |  |  |
| Crude model |  |  |  |  |  |  |  |  |  |  |  |  |
| 8.3 – <16.7 | vs | <8.3 | -0.06 | (-0.39 – 0.26) | 1.00 |  | 0.03 | (-0.42 – 0.47) | 1.00 | 0.7481 | 0.6613 | 0.2545 |
| 16.7 – <33.3 | vs | <8.3 | 0.01 | (-0.28 – 0.31) | 1.00 |  | 0.18 | (-0.31 – 0.67) | 1.00 |  |  |  |
| 33.3 – <50 | vs | <8.3 | -0.12 | (-0.42 – 0.18) | 1.00 |  | 0.07 | (-0.39 – 0.52) | 1.00 |  |  |  |
| ≥50 | vs | <8.3 | -0.14 | (-0.42 – 0.15) | 1.00 |  | 0.02 | (-0.41 – 0.46) | 1.00 |  |  |  |
|  |  |  |  |  |  |  |  |  |  |  |  |  |
| Fully adjusted model | | |  |  |  |  |  |  |  |  |  |  |
| 8.3 – <16.7 | vs | <8.3 | -0.06 | (-0.39 – 0.26) | 1.00 |  | 0.03 | (-0.41 – 0.47) | 1.00 | 0.7121 | 0.5576 | 0.2568 |
| 16.7 – <33.3 | vs | <8.3 | 0.01 | (-0.29 – 0.3) | 1.00 |  | 0.18 | (-0.31 – 0.68) | 1.00 |  |  |  |
| 33.3 – <50 | vs | <8.3 | -0.14 | (-0.44 – 0.17) | 1.00 |  | 0.06 | (-0.39 – 0.51) | 1.00 |  |  |  |
| ≥50 | vs | <8.3 | -0.15 | (-0.43 – 0.14) | 1.00 |  | 0.02 | (-0.42 – 0.46) | 1.00 |  |  |  |

*LTPA = leisure-time physical activity; SD = standard deviation; CI = confidence interval*

*Crude model: adjusted for sex and age.*

*Fully adjusted model: adjusted for sex, age, chronic diseases, smoking, alcohol usage, socioeconomic status, follow-up time, body mass index and medication.*
